# Maternal dietary intake of fish and child neurodevelopment at 3 years: a nationwide birth cohort—The Japan Environment and Children's Study

**DOI:** 10.3389/fpubh.2023.1267088

**Published:** 2024-01-24

**Authors:** Mariko Inoue, Kenta Matsumura, Kei Hamazaki, Akiko Tsuchida, Hidekuni Inadera

**Affiliations:** ^1^Department of Public Health, Faculty of Medicine, University of Toyama, Toyama, Japan; ^2^Toyama Regional Center for JECS Study, Faculty of Medicine, University of Toyama, Toyama, Japan; ^3^Department of Public Health, Gunma University Graduate School of Medicine, Maebashi, Japan

**Keywords:** pregnancy, fish, neurodevelopment, infant, food life

## Abstract

**Background:**

Results on the association between fish intake during pregnancy and a reduction in neurodevelopmental delays in children have been inconsistent, with some reports finding an association and others finding none. Because neurodevelopmental delays are more pronounced at the age of 3 years, their association needs to be examined at this age.

**Methods:**

After exclusion and multiple imputation from a dataset comprising 104,057 records from the Japan Environment and Children's Study, logistic regression analysis was conducted in quintiles to evaluate the association between maternal fish intake during pregnancy and child neurodevelopment at age 3 years in 91,909 mother–child pairs. The Food Frequency Questionnaire (FFQ), validated in the Japan Public Health Center-Based Prospective Study for the Next Generation, was used to assess maternal fish intake during pregnancy. The Ages and Stages Questionnaires-3 was used to assess children's neurodevelopment in five domains: communication, gross motor, fine motor, problem-solving, and personal-social.

**Results:**

Consistently lower odds were found for the highest vs. lowest quintile for the domains of communication, fine motor, problem-solving, and personal-social but not gross motor skills, with adjusted odd ratios (95% confidence intervals) of 0.89 (0.80–0.998), 0.90 (0.83–0.97), 0.86 (0.80–0.94), 0.87 (0.77–0.98), and 1.04 (0.94–1.16), respectively. The trend for lower odds of symptoms of neurodevelopmental delays across quintiles of higher maternal fish intake were significant for fine motor, problem-solving, and personal-social but not communication or gross motor.

**Conclusions:**

Fish consumption during pregnancy may be associated with a reduced risk of neurodevelopmental delay in 3-year-olds, particularly in the fine motor, problem-solving, and personal-social domains. Continued investigation after the age of 3 could further clarify the association.

## 1 Introduction

Maternal fish intake during pregnancy has been associated with fewer adverse birth events, especially preterm birth (1), and a lower risk of wheeze, eczema, and food allergy in offspring (2). On the other hand, the relationship between maternal fish intake during pregnancy and child neurodevelopment is controversial (3–6).

Studies linking maternal seafood intake to child neurodevelopment have reported that low maternal seafood intake increases the risk of suboptimal outcomes in children's prosocial behavior, fine motor, communication, and social development scores (3) and that maternal seafood intake during pregnancy is associated with better neurodevelopment at 14 months and 5 years of age (7). A large study of 10,026 mothers also reported that maternal omega-3 docosahexaenoic acid (DHA) intake during pregnancy was positively correlated with infant DHA status at 3 months of age and with problem-solving ability at 12 months of age, that children whose mothers had higher levels of DHA had better language development, and that the positive effects of omega-3 fatty acids in seafood and n-3 polyunsaturated fatty acid (PUFA) intake during pregnancy outweighed the negative effects of mercury (8). However, some studies have shown that seafood and n-3 PUFA intake during pregnancy is not associated with neurodevelopment in children, particularly studies conducted in Japan. For example, no significant association was found between total seafood intake during pregnancy and motor cluster scores on the Neonatal Behavior Rating Scale for 3-day-old infants (5) and no significant association was identified between maternal n-3 PUFA fatty acid intake during pregnancy and development of attention-deficit/hyperactivity disorder (ADHD) at age 5 years (6).

Previously, we hypothesized that fish consumption by mothers during pregnancy might enhance psychomotor development in their children, and we examined this association in children at 6 months and 1 year of age (4). Using data from the Japan Environment and Children's Study (JECS), a nationwide survey, we assessed 81,697 and 77,751 mother–child pairs at 6 months and 1 year of age, respectively. The results showed that maternal fish intake during pregnancy was independently associated with a reduced risk of delay in problem-solving skills at 6 months of age and in fine motor and problem-solving skills at 1 year of age.

The inconsistency in results thus far may have been influenced by regional and cultural differences in fish consumption. For example, in a Japanese study, Miyake et al. (6) suggested that the reason they did not find an association between maternal eicosapentaenoic acid (EPA) or DHA intake and child behavioral problems was their study population lived in areas with high fish consumption and speculated that an association might be found in populations with low fish consumption. In our previous study, the median intake of fish by pregnant mothers was 29.9 g/day (4), which is low compared with studies reporting no association (5, 9). Given the declining trend in fish consumption in the Japanese population (10), it is important to study the long-term effects of mothers' fish consumption during pregnancy on their children's neurodevelopment in large populations.

It has been reported that developmental delays are innate (11) and gradually become more pronounced by the age of 3 years (12). Specifically, by that age, children are able to use the fingers of both hands and are able to understand the meaning of words and express their intentions (13). In addition, the ability to decide on one's own actions nears completion at the age of 3–4 years (13). For this reason, in Japan, mental developmental tests have been introduced into examinations in early childhood (14), and the importance of the early detection and follow-up of developmental delays during limited screening has been reported (15). In particular, the age between 3 and 4 years is expected to be a particularly important developmental stage because of the increase in social activities preceding compulsory education, such as kindergarten attendance.

Therefore, the purpose of this study was to clarify the relationship between fish intake during pregnancy and neurodevelopment at age 3 years and also to focus on the neurodevelopmental associations observed at 6 months and 1 year of age and those observed at age 3 years in a large nationwide population.

## 2 Methods

### 2.1 Study population

The JECS protocol has been described in detail elsewhere (16). Briefly, the aim of the JECS, a nationwide government-funded birth cohort study, is to evaluate the impact of various environmental factors on child health and development. Between January 2011 and March 2014, participants (expectant mothers) were recruited face to face by cooperating healthcare providers at 15 regional centers, covering rural and urban areas, throughout Japan. Eligibility criteria for the participants were as follows: (1) resident in a study area at the time of recruitment and expecting to continue living in Japan for the foreseeable future; (2) expected delivery date between 1 August 2011 and mid-2014; and (3) ability to comprehend the Japanese language and complete the self-administered questionnaires. We excluded those residing outside of a study area even if they visited a cooperating healthcare provider within a study area. The participation acceptance rate was 78.5%.

Data pertaining to the following periods were collected on nine occasions up until 3 years after delivery: during early pregnancy (mean ± SD: 16.7 ± 7.7 weeks of gestation), during mid-late pregnancy (mean ± SD: 28.2 ± 6.5 weeks of gestation), and at 1, 6, 12, 18, 24, 30, and 36 months after delivery. Mothers completed a self-administered questionnaire on demographic characteristics, socioeconomic status, diet, child development, and other topics at those times. JECS staff distributed and collected the questionnaire for the first three timepoints during the participants' health check-ups at the maternity hospital up until 1 month after delivery. When collection was not possible at these times, questionnaires were returned by mail. Subsequent questionnaires were distributed and collected by mail.

**Figure 1:**
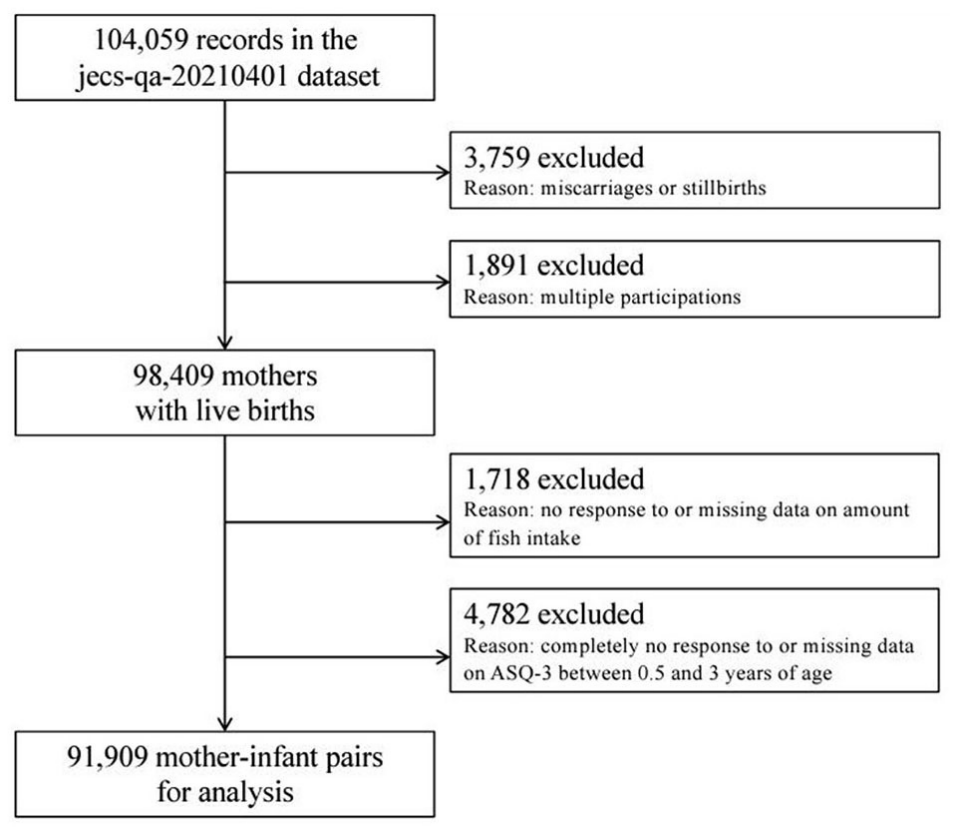
Participants flow chart.

The present study is based on the JECS-QA-20210401 dataset, which was released in April 2021. The full dataset comprises 104,059 records, but we excluded 3759 records because of miscarriages/stillbirths and 1,891 records because of multiple births (Figure 1). We also excluded 1,718 mothers because of no response or missing data on fish intake and 4,782 mothers because of no response or missing data for the ASQ-3 (Ages and Stages Questionnaires^®^, Third Edition). This left data for 91,909 mother–child pairs for analysis.

The study protocol was reviewed and approved by the Ministry of the Environment's Institutional Review Board on Epidemiological Studies and the ethics committees of all participating institutions. All participants provided written informed consent.

### 2.2 Measurements of fish intake

Maternal fish intake during pregnancy was measured using the food frequency questionnaire (FFQ). The FFQ is a semiquantitative instrument that consists of three components: list of food items, average frequency of intake, and average portion size (average intake per serving). For each food item, the average frequency is multiplied by the average portion size, and the food items can be summed within a category (e.g., fish intake) in order to estimate average dietary intake over a period of several months from a single questionnaire. The FFQ has been validated for use in large-scale Japanese epidemiological studies (17). It covers more than 170 food and beverage items, including 21 related to fish or shellfish, and it has nine frequency categories ranging from “almost never” to “seven or more times per day” (or “10 or more glasses per day” for beverages) and three portion size categories of small (50% smaller than standard), medium (same as standard), or large (50% larger than standard).

Participants answered how often and how much they consumed each food type during mid-late pregnancy (average dietary intake after learning of the pregnancy to the time of questionnaire completion). For each of the 21 fish or shellfish items, the common 9 frequency categories for each item of < 1 time/month, 1–3 times/month, 1–2 times/week, 3–4 times/week, 5–6 times/week, every day, 2–3 times/day, 4–6 times/day, and ≥7 times/day were used. The standard portion size (with the approximate equivalent size in grams) of each of the 21 fish or shellfish items listed was as follows: slice of salted fish (70 g); one whole dried fish (50 g); quarter of a can of tuna (20 g); slice of salmon or trout (70 g); four sashimi slices of bonito or tuna (60 g);four sashimi slices of Japanese amberjack (60 g); half slice of cod or flatfish (40 g); slice of sea bream (70 g); one whole horse mackerel or sardine (80 g); one whole saury or mackerel (80 g); one tablespoons of small dried fish (10 g); one-quarter of a clutch of salted roe (20 g); half skewer of eel (50 g); three sashimi slices of squid (50 g); one-third of an octopus tentacle (50 g); two Chinese white shrimps (40 g); 10 shucked clams (20 g); 10 shucked pond snails (20 g); and fish paste products comprising one-sixth of chikuwa (20 g); two slices of kamaboko (20 g), and one-quarter of satsuma-age (20 g).

Epidemiological studies have reported that total energy intake is often associated with disease risk because of the association between physical activity and body size and the likelihood of disease occurrence. In other words, to control for confounding, reduce ambulatory variation, and predict the effects of dietary interventions, epidemiological studies usually recommend adjusting for total energy intake, so fish intake was log-transformed and energy-adjusted intake was calculated using residual mode (18). Because some participants had fish intake of 0 g/day, we replaced this value with 0.03 g/day, which is one-tenth of the lowest fish intake (0.3 g/day) of all participants (excluding 0 g/day).

### 2.3 Neurodevelopment

The quantitative use of the Ages and Stages Questionnaires^®^, Third Edition (ASQ-3), a parent-completed method for screening children at risk of developmental delay in children between the ages of 1 month and 5$\frac{1}{2}$ years, has been validated in epidemiological studies (19). The Japanese version of the ASQ-3 has also been validated (20) and has been used in several studies (21, 22). The ASQ-3 assesses the following 5 developmental domains: (a) communication: items related to language skills, such as babbling, vocalizing, listening, and understanding, including being able to answer to their own name and give simple directions using words; (b) gross motor: items related to arm, body, and leg movements during movement and play, such as being able to kick a ball and jump; (c) fine motor: items measuring hand and finger movements, such as the ability to use scissors to cut paper and to use pencils and crayons appropriately; (d) problem-solving: items related to problem-solving skills, learning, and playing with toys, such as reciting numbers and being able to arrange blocks in the same way as demonstrated; and (e) personal-social: items related to self-help skills, solitary social play, and play with toys and others, such as being able to dress oneself and keep order.

In total, the ASQ-3 comprises 30 questions (6 per domain) that can be answered with either “yes” (=10), “sometimes” (=5), or “not yet” (=0), resulting in a score of 0–60 for each domain. If 1 or 2 answers out of the 6 questions were missing for a domain, the total score for the items answered was multiplied by a correction coefficient of 1.2 or 1.5, respectively, to adjust the score to 0–60. If >2 of the 6 questions were not answered, the participant was excluded from the analysis. The timing for administering the ASQ-3 at 3 years after delivery was set to within ±1 month (from 35 months 0 days to 36 months 30 days), and participants who completed it outside of this period were excluded. Age was corrected for prematurity if a child was born ≥3 weeks before the due date. Screen-positive cases for each domain were defined as those with scores at or below the cut-offs for (a) communication: 29.95, (b) gross motor: 39.26, (c) fine motor: 27.91, (d) problem-solving: 30.03, and (e) personal-social: 29.89 (20).

### 2.4 Statistical analysis

To determine the association between overall fish intake and children's development at 3 years old, quintiles of fish intake during pregnancy were calculated, following previous studies (4, 23). We then calculated odds ratios (ORs) and 95% confidence intervals (CIs) using logistic regression analysis, with the 15 regional centers set as a random effect. In tests for trend, we assigned categorical numbers to the quintile distributions for fish intake and evaluated them as continuous variables. We included potential confounding factors and covariates in the statistical analysis if previous studies found them to be associated (or they are theoretically inferred to be associated) with the outcome. These were assessed by the questionnaires administered during early and mid–late pregnancy. Because birth weight, gestation length, and breastfeeding are post-exposure covariates and are considered mediators not confounders, we did not adjust for these covariates. The confounding factors and covariates used in this study were as follows: age (years, continuous variable); physical activity [METs·h/day, measured using the International Physical Activity Questionnaire (24, 25), continuous variable]; previous deliveries (nulliparous or multiparous); pre-pregnancy body mass index (BMI; kg/m^2^), categorized as < 18.5, 18.5–25, or ≥25; highest maternal education level (1, junior high or high school; 2, technical junior college, technical/vocational college, or associate degree; or 3, bachelor's degree or postgraduate degree); annual household income (< 4 million, 4–6 million, or ≥6 million JPY); marital status [1, married (including common-law status); 2, single (never married); or 3, divorced or widowed]; alcohol intake (1, never; 2, previously drank alcohol but quit before learning of the pregnancy; 3, previously drank alcohol but quit after learning of the pregnancy; or 4, currently drinking; because 5 participants were misclassified as belonging to category No. 5 “quit,” which was not an option, we treated them as having missing data and later imputed them using multiple imputation); smoking status (1, never; 2, previously smoked but quit before learning of the pregnancy; 3, previously smoked but quit after learning of the pregnancy; or 4, currently smoking); employment status (yes or no); child's sex (female or male); presence of a major congenital anomaly at delivery and at age 1 month (26), yes or no; and use of EPA and/or DHA supplementation (yes for ≥1–3 uses/week or no for ≤ 2–3 uses/month); and maternal psychological distress [Kessler Psychological Distress Scale (27–29) score ≥5 or not].

We performed multiple imputations for the missing values of covariates by using chained equations (30) to obtain 10 imputed datasets. We included auxiliary variables related to covariates to preserve the assumption of missing at random. Statistical significance was set at a 2-sided *p* < 0.05. Analyses were performed with SAS version 9.4 (SAS Institute Inc).

### 2.5 Additional analysis

To assess the robustness of the results, ORs were also calculated for the lowest tertile and quartile, using multivariate logistic regression analysis.

## 3 Results

### 3.1 Participant characteristics

Table 1 shows the maternal characteristics of the participants according to quintile of fish intake. Women who reported eating more fish were slightly older, more likely to have multiple children, have higher educational attainment and annual household income, be unemployed, and be a non-smoker compared with women who reported eating less fish.

**Table 1:** Characteristics of participants according to quintile of fish intake (*N* = 91,909).

		**Quintile for fish intake** ^**a**^										
		**1 (low)**		**2**		**3**		**4**		**5 (high)**		
		**(*****n** =* **18,381)**		**(*****n** =* **18,382)**		**(*****n** =* **18,382)**		**(*****n** =* **18,382)**		**(*****n** =* **18,382)**		
		**n**	**(%)**	**n**	**(%)**	**n**	**(%)**	**n**	**(%)**	**n**	**(%)**	*p* ^*^
Median intake of fish^a^, g/day		5.2		18.4		30.0		43.8		70.4		
Mother's age at childbirth, y	Mean ± SD	30.5	± 1.6	31.2	± 1.6	31.4	± 1.6	31.7	± 1.5	31.5	± 1.6	
Physical activity, METs·h/day	Mean ± SD	4.5	± 3.0	4.0	± 2.6	3.9	± 2.6	3.7	± 2.4	3.8	± 2.5	
Parity	Primipara	8,473	(46.1)	7,504	(40.8)	7,273	(39.6)	7,140	(38.8)	7,409	(40.3)	**< 0.001**
	Multipara	9,909	(53.9)	10,878	(59.2)	11,109	(60.4)	11,242	(61.2)	10,973	(59.7)	
Body mass index, kg/m^2^	< 18.5	3,011	(16.4)	2,965	(16.1)	2,916	(15.9)	2,950	(16.1)	3,005	(16.4)	**< 0.001**
	18.5– < 25	13,399	(72.9)	13,602	(74.0)	13,690	(74.5)	13,474	(73.3)	13,348	(72.6)	
	≥25	1,971	(10.7)	1,815	(9.9)	1,777	(9.7)	1,958	(10.7)	2,029	(11.0)	
Highest education level, y	≤ 12	7,723	(42.0)	6,533	(35.5)	6,137	(33.4)	5,867	(31.9)	6,433	(35.0)	**< 0.001**
	>12– ≤ 16	7,525	(40.9)	7,924	(43.1)	7,951	(43.3)	7,909	(43.0)	7,634	(41.5)	
	>16	3,133	(17.0)	3,926	(21.4)	4,294	(23.4)	4,606	(25.1)	4,315	(23.5)	
Annual household income, JPY	< 4 million	8,590	(46.7)	7,475	(40.7)	7,207	(39.2)	6,781	(36.9)	6,881	(37.4)	**< 0.001**
	4– < 6 million	5,679	(30.9)	6,127	(33.3)	6,161	(33.5)	6,201	(33.7)	6,259	(34.1)	
	≥6 million	4,112	(22.4)	4,781	(26.0)	5,015	(27.3)	5,401	(29.4)	5,241	(28.5)	
Marital status	Married	17,267	(93.9)	17,627	(95.9)	17,725	(96.4)	17,763	(96.6)	17,641	(96.0)	**< 0.001**
	Unmarried	893	(4.9)	601	(3.3)	539	(2.9)	504	(2.7)	603	(3.3)	
	Divorced/widowed	221	(1.2)	154	(0.8)	118	(0.6)	115	(0.6)	138	(0.8)	
Alcohol intake	Never	6,286	(34.2)	6,040	(32.9)	6,213	(33.8)	6,024	(32.8)	6,300	(34.3)	**< 0.001**
	Quit before learning of pregnancy	3,092	(16.8)	3,139	(17.1)	3,085	(16.8)	3,284	(17.9)	3,137	(17.1)	
	Quit after learning of pregnancy	8,546	(46.5)	8,656	(47.1)	8,526	(46.4)	8,548	(46.5)	8,468	(46.1)	
	Current	457	(2.5)	548	(3.0)	558	(3.0)	526	(2.9)	476	(2.6)	
Smoking status	Never	9,770	(53.2)	10,633	(57.8)	11,040	(60.1)	11,253	(61.2)	10,978	(59.7)	**< 0.001**
	Quit before learning of pregnancy	4,501	(24.5)	4,530	(24.6)	4,357	(23.7)	4,350	(23.7)	4,322	(23.5)	
	Quit after learning of pregnancy	3,074	(16.7)	2,445	(13.3)	2,253	(12.3)	2,149	(11.7)	2,359	(12.8)	
	Current	1,036	(5.6)	775	(4.2)	732	(4.0)	630	(3.4)	723	(3.9)	
Employment status	No	8,067	(43.9)	8,385	(45.6)	8,459	(46.0)	8,636	(47.0)	8,801	(47.9)	**< 0.001**
	Yes	10,314	(56.1)	9,997	(54.4)	9,923	(54.0)	9,746	(53.0)	9,581	(52.1)	
Child's sex	Girls	8,968	(48.8)	8,970	(48.8)	8,957	(48.7)	8,923	(48.5)	9,010	(49.0)	0.932
	Boys	9,413	(51.2)	9,412	(51.2)	9,425	(51.3)	9,459	(51.5)	9,372	(51.0)	
Congenital anomaly	No	17,976	(97.8)	17,956	(97.7)	17,999	(97.9)	17,970	(97.8)	17,948	(97.6)	0.420
	Yes	405	(2.2)	426	(2.3)	383	(2.1)	412	(2.2)	434	(2.4)	
Use of EPA/DHA supplementation	No	17,890	(97.3)	17,914	(97.5)	17,927	(97.5)	17,925	(97.5)	17,860	(97.2)	0.152
	Yes	491	(2.7)	468	(2.5)	455	(2.5)	457	(2.5)	522	(2.8)	
Psychological distress	No	12,538	(68.2)	13,017	(70.8)	13,174	(71.7)	13,484	(73.4)	13,546	(73.7)	**< 0.001**
	Yes	5,843	(31.8)	5,365	(29.2)	5,208	(28.3)	4,899	(26.7)	4,836	(26.3)	

Bold indicates significance. ^*^Derived from logistic regression analysis–assigned categorical numbers to the quintile distributions and evaluated as continuous variables. ^a^Energy-adjusted average dietary intake for the period after participants learned of the pregnancy up until mid-late pregnancy.

### 3.2 Multivariable analysis of psychomotor development domains at age 3 years

Table 2 shows the multivariable ORs (with 95% CIs) for the scoring of each psychomotor development domain at age 3 according to quintile of fish intake during pregnancy (*n* = 91,909). The results revealed reduced risks of delay at 3 years in the second and fifth quintiles for communication (0.89 [0.80–0.996] and 0.89 [0.80–0.998]), in the fourth and fifth quintiles for fine motor (0.89 [0.82–0.97] and 0.90 [0.83–0.97]), in the second, third, fourth, and fifth quintiles for problem-solving (0.90 [0.83–0.98], 0.87 [0.80–0.94], 0.89 [0.82–0.96], and 0.86 [0.80–0.94]), and in the second, fourth, and fifth quintiles for personal-social (0.88 [0.78–0.999], 0.88 [0.78–0.998], and 0.87 [0.77–0.98]). A trend test also revealed a significant linear association of fish intake with fine motor, problem-solving, and personal-social at age 3 years. However, communication and gross motor showed no significant results in the trend test, and gross motor showed no differences between groups.

**Table 2:** Multivariable analysis for psychomotor development domains at age 3 years according to quintile of fish intake during pregnancy (*N* = 91,909).

		**Quintile for fish intake** ^**a**^					*p*-value for trend ^*^
		**1 (low)**	**2**	**3**	**4**	**5 (high)**	
Communication	Crude OR	1.00 —	**0.88 (0.79–0.98)**	**0.89 (0.80–0.99)**	0.90 (0.81–1.01)	0.90 (0.81–1.01)	0.126
	Adjusted OR	1.00 —	**0.89 (0.80–0.996)**	0.90 (0.80–1.002)	0.91 (0.81–1.01)	**0.89 (0.80–0.998)**	0.094
Gross motor	Crude OR	1.00 —	1.00 (0.90–1.11)	0.97 (0.87–1.08)	1.00 (0.89–1.11)	1.07 (0.97–1.19)	0.237
	Adjusted OR	1.00 —	0.99 (0.89–1.10)	0.96 (0.86–1.06)	0.97 (0.87–1.08)	1.04 (0.94–1.16)	0.573
Fine motor	Crude OR	1.00 —	0.97 (0.90–1.05)	**0.90 (0.83–0.98)**	**0.85 (0.78–0.92)**	**0.87 (0.80–0.94)**	**< 0.001**
	Adjusted OR	1.00 —	1.01 (0.93–1.09)	0.94 (0.87–1.02)	**0.89 (0.82–0.97)**	**0.90 (0.83–0.97)**	**< 0.001**
Problem-solving	Crude OR	1.00 —	**0.90 (0.83–0.98)**	**0.86 (0.79–0.94)**	**0.89 (0.82–0.96)**	**0.87 (0.80–0.95)**	**0.002**
	Adjusted OR	1.00 —	**0.90 (0.83–0.98)**	**0.87 (0.80–0.94)**	**0.89 (0.82–0.96)**	**0.86 (0.80–0.94)**	**0.001**
Personal-social	Crude OR	1.00 —	**0.87 (0.77–0.98)**	**0.88 (0.78–0.99)**	**0.88 (0.78–0.99)**	**0.87 (0.78–0.98)**	**0.049**
	Adjusted OR	1.00 —	**0.88 (0.78–0.999)**	0.89 (0.79–1.01)	**0.88 (0.78–0.998)**	**0.87 (0.77–0.98)**	**0.037**

Bold indicates significance. ^*^Derived from logistic regression analysis–assigned categorical numbers to the quintile distributions and evaluated as continuous variables. Covariates were adjusted for mother's age, previous deliveries, pre-pregnancy BMI (kg/m^2^), highest maternal education level, annual household income, marital status, alcohol intake, smoking status, employment status, child's sex, presence of a congenital anomaly, use of EPA and/or DHA supplementation, and psychological distress. ^a^Energy-adjusted average dietary intake for the period after participants learned of the pregnancy up until mid-late pregnancy.

In the additional analysis, ORs were calculated for fish intake during pregnancy and children's development at 3 years of age in both the lowest third and fourth quartiles. There were consistently lower odds in the highest vs. lowest tertile and quartile for fine motor and problem-solving but not for communication, gross motor, or personal-social skills. The trend for lower odds of symptoms of neurodevelopmental delay across tertiles or quartiles of higher maternal fish intake were significant for fine motor and problem-solving but not for communication, gross motor, or personal-social skills (Supplementary Tables 1, 2), suggesting a consistent dose-effect relationship, at least for fine motor and problem solving, regardless of the method of analysis.

## 4 Discussion

This study used data from 91,909 mother–child pairs from the JECS to determine the association of the dietary intake of fish during pregnancy with neurodevelopment in 3-year-old children. The results showed reduced risks of a communication delay for the second and fifth quintiles, of a fine motor delay for the fourth and fifth quintiles, of a problem-solving delay for the second, third, fourth, and fifth quintiles, and of a personal-social delay for the second, fourth, and fifth quintiles. Gross motor showed no significant results in the trend test or differences between groups.

Studies focusing on the association between maternal seafood intake and children's neurodevelopment have reported that maternal seafood intake is associated with the improved neuropsychological development of children at 14 months and, in particular, at 5 years of age (7). In addition, studies focusing on children's fish intake have reported that children with high fish intake have a higher sleep quality and intelligence quotient than those with low intake (31, 32). On the other hand, other reports failed to find an association of maternal seafood intake during pregnancy with children's neurodevelopment (33, 34). These reports were more commonly randomized controlled studies than observational studies, suggesting the presence of limitations such as an inability to actively reduce nutrient intake for ethical reasons, inability to evaluate long-term interventions, and inability to intervene in fish intake as a whole food (4). Indeed, many randomized controlled studies in animals have reported positive results.

Randomized controlled trials in animals have suggested that high levels of PUFAs, which are abundant in fish, have positive effects on development: rats with sufficient n-3 fatty acids have better memory learning ability than rats lacking n-3 fatty acids (35). In addition, it has been shown that DHA deficiency during brain maturation in rats impairs brain function in adulthood and that adequate amounts of DHA are important for long-term neuronal resilience in the brain (36).

Fish is rich in nutrients important for infant development, such as iodine and vitamins A, D, and B12 (37, 38), and has been found to function as a proxy for a healthy lifestyle (38). Given that common chronic diseases are potentially preventable when nutritional status is optimized for the fetus (39), it is expected that high fish intake during pregnancy will have a positive impact on children's neurodevelopment. However, the results of the present study showed no differences in trend tests or group ratings for gross motor activity. This was similar to the results of previous studies conducted at 6 months and 1 year of age (4), and suggests that fish consumption during pregnancy may have a positive effect on some aspects of neurodevelopment in children but may not be associated with gross motor activity. However, more research is needed because gross motor skills such as one-legged kneeling and swinging alone continue to develop after 3 years of age.

The strengths of this study are the size of the cohort, that the participants are likely to be representative of the Japanese population (40), and that the dataset is extensive, which allowed us to adjust for a large number of covariates. In addition, the ASQ-3 questionnaire used in this study as a screening tool for monitoring children who are at risk of developmental delay is well validated for quantitative use in epidemiological studies and is used worldwide (41). The prospective data collection of exposures and outcomes minimized recall bias, and almost all important confounders were included in the model. Moreover, the FFQ contains as many as 21 items related to fish or shellfish consumption (17).

However, the study also has several limitations that should be considered. First, the FFQ has been validated (17) but not specifically for pregnant women. Second, as noted above, due to the observational nature of the study, the results may have been confounded by unmeasured residual factors. This is one of the major drawbacks of observational studies. It was not possible to consider all other dietary intakes and dietary patterns. For example, fish consumption may serve as a proxy for an overall healthy lifestyle (38), and people may choose nutrient-rich options more frequently than nutritionally unbalanced and/or nutrient-deficient options, such as junk food. Furthermore, there is also geographical variation in the amount and type of fish and shellfish consumed, and shellfish in particular is reported to be affected by environmental pollution (42). In Japan, “Precautions for pregnant women regarding ingestion of fish and shellfish and mercury” that appears on the Ministry of Health, Labor and Welfare website advises caution when eating fish that may contain high levels of mercury, such as bigeye tuna, salmon, and yellowtail (43). This study did not take into account factors such as the origin of the fish consumed, the method of storage and freshness of the fish, the method of cooking, or the amount of contaminants in the fish consumed, which should be examined in future work. Third, the FFQ responses are based on the mother's memory, so recall bias cannot be ruled out, and the ASQ-3 findings are also based on parental reports and not an objective assessment. Fourth, this study dealt only with maternal fish intake during pregnancy and could not consider the children's own intake. Fifth, although we covered both rural and urbans locations across Japan in this national study, the participants may not necessarily have been a representative sample of Japanese pregnant women. Nevertheless, the basic characteristics of the mothers, fathers, and children participating in the JECS are very close to those of the Japanese government's Vital Statistics, and the JECS data are considered to reflect the birth situation in Japan. Nevertheless, even if the participants in this study were a representative sample of pregnant Japanese women, who generally consume more fish than their Western counterparts, the findings may not be generalizable to Western populations.

## 5 Conclusions

Fish consumption during pregnancy may be associated with a reduced risk of delay in the neurodevelopment of 3-year-olds, particularly in the fine motor, problem-solving, and personal-social domains. Continued investigation after the age of 3 could further clarify the association.

## Data availability statement

Data are unsuitable for public deposition due to ethical restrictions and legal framework of Japan. It is prohibited by the Act on the Protection of Personal Information (Act No. 57 of 30 May 2003, amended 9 September 2015) to publicly deposit data containing personal information. Ethical Guidelines for Medical and Health Research Involving Human Subjects enforced by the Japan Ministry of Education, Culture, Sports, Science and Technology and the Ministry of Health, Labour and Welfare also restrict the open sharing of epidemiologic data. All inquiries about access to data should be sent to: jecs-en@nies.go.jp. The person responsible for handling enquiries sent to this e-mail address is Dr. Shoji F. Nakayama, JECS Programme Office, National Institute for Environmental Studies.

## Ethics statement

The studies involving humans were approved by The Ministry of the Environment's Institutional Review Board on Epidemiological Studies and the Ethics Committees of all participating institutions. The studies were conducted in accordance with the local legislation and institutional requirements. Written informed consent for participation in this study was provided by the participants' legal guardians/next of kin.

## Author contributions

MI: Conceptualization, Writing – original draft. KM: Formal analysis, Writing – review & editing. KH: Conceptualization, Investigation, Supervision, Writing – review & editing. AT: Investigation, Writing – review & editing. HI: Investigation, Project administration, Writing – review & editing.

## Members of the JECS Group as of 2022

Michihiro Kamijima (Principal Investigator, Nagoya City University, Nagoya, Japan), Shin Yamazaki (National Institute for Environmental Studies, Tsukuba, Japan), Yukihiro Ohya (National Center for Child Health and Development, Tokyo, Japan), Reiko Kishi (Hokkaido University, Sapporo, Japan), Nobuo Yaegashi (Tohoku University, Sendai, Japan), Koichi Hashimoto (Fukushima Medical University, Fukushima, Japan), Chisato Mori (Chiba University, Chiba, Japan), Shuichi Ito (Yokohama City University, Yokohama, Japan), Zentaro Yamagata (University of Yamanashi, Chuo, Japan), Hidekuni Inadera (University of Toyama, Toyama, Japan), Takeo Nakayama (Kyoto University, Kyoto, Japan), Tomotaka Sobue (Osaka University, Suita, Japan), Masayuki Shima (Hyogo Medical University, Nishinomiya, Japan), Hiroshige Nakamura (Tottori University, Yonago, Japan), Narufumi Suganuma (Kochi University, Nankoku, Japan), Koichi Kusuhara (University of Occupational and Environmental Health, Kitakyushu, Japan), and Takahiko Katoh (Kumamoto University, Kumamoto, Japan).
