# Supplementary material for: Maternal dietary intake of fish and child neurodevelopment at 3 years: a nationwide birth cohort—The Japan Environment and Children's Study
Source: Front Public Health. 2024 Jan 24;11:1267088. doi: 10.3389/fpubh.2023.1267088 (PMC10847349; doi:10.3389/fpubh.2023.1267088)
Supplement: Supplementary file 1 [file Table_1.docx]

**Supplemental Table 1.** Adjusted odds ratios for the multivariable analysis of psychomotor development domains at age 3 years according to tertile of fish intake during pregnancy (*N* = 91,909)

|  | Tertile for fish intake^1^ | |  | *p*-value  for trend * |
| --- | --- | --- | --- | --- |
|  | 1 (low) | 2 | 3 |  |
| Communication | 1.00 –– | 0.97 (0.89–1.05) | 0.92 (0.84–1.00) | 0.064 |
| Gross motor | 1.00 –– | 1.02 (0.93–1.11) | 1.03 (0.95–1.12) | 0.427 |
| Fine motor | 1.00 –– | 0.96 (0.90–1.02) | **0.89 (0.84–0.95)** | **< 0.001** |
| Problem-solving | 1.00 –– | **0.93 (0.88–1.00)** | **0.91 (0.85–0.97)** | **0.003** |
| Personal-social | 1.00 –– | 0.97 (0.89–1.07) | 0.92 (0.84–1.02) | 0.106 |
| Bold indicates significance.  Covariates were adjusted for mother’s age, previous deliveries, pre-pregnancy BMI (kg/m^2^), highest maternal education level,  annual household income, marital status, alcohol intake, smoking status, employment status, child’s sex, presence of a congenital anomaly, use of EPA and/or DHA supplementation, and psychological distress.  ^1^ Energy-adjusted average dietary intake for the period after participants they learned of the pregnancy up until mid-late pregnancy. | | | | |

^＊^Derived from logistic regression analysis assigned–categorical numbers to the quintile distributions and evaluated as continuous variables.

**Supplemental Table 2.** Adjusted odds ratios for multivariable analysis of psychomotor development domains at age 3 years according to quartile of fish intake during pregnancy (*N* = 91,909)

|  | Quartile for fish intake^1^ | |  |  | *p*-value  for trend * |
| --- | --- | --- | --- | --- | --- |
|  | 1 (low) | 2 | 3 | 4 (high) |  |
| Communication | 1.00 –– | 0.94 (0.85–1.03) | 0.95 (0.86–1.05) | 0.92 (0.83–1.01) | 0.136 |
| Gross motor | 1.00 –– | 1.01 (0.92–1.11) | 1.01 (0.92–1.12) | 1.05 (0.95–1.15) | 0.329 |
| Fine motor | 1.00 –– | 1.01 (0.94–1.08) | **0.92 (0.85–0.99)** | **0.89 (0.83–0.96)** | **< 0.001** |
| Problem-solving | 1.00 –– | 0.93 (0.86–1.00) | **0.92 (0.86–0.99)** | **0.89 (0.82–0.96)** | **0.002** |
| Personal-social | 1.00 –– | 0.93 (0.83–1.03) | 0.96 (0.86–1.08) | 0.94 (0.84–1.04) | 0.351 |
| Bold indicates significance.  Covariates were adjusted for mother’s age, previous deliveries, pre-pregnancy BMI (kg/m^2^), highest maternal education level,  annual household income, marital status, alcohol intake, smoking status, employment status, child’s sex, presence of a congenital anomaly, use of EPA and/or DHA supplementation, and psychological distress.  ^1^ Energy-adjusted average dietary intake for the period after participants learned of the pregnancy up until mid-late pregnancy. | | | | | |

^＊^Derived from logistic regression analysis–assigned categorical numbers to the quintile distributions and evaluated as continuous variables.
